# An open-source remote heart rate imaging method with practical apparatus and algorithms

**DOI:** 10.3758/s13428-019-01256-8

**Published:** 2019-05-31

**Authors:** Koen M. van der Kooij, Marnix Naber

**Affiliations:** Experimental Psychology, Faculty of Social Sciences, Utrecht University, Utrecht, The Netherlands

**Keywords:** Heart rate, Remote photoplethysmography, Pulse oximetry, Respiration, Exercise

## Abstract

Recent developments in computer science and digital image processing have enabled the extraction of an individual’s heart pulsations from pixel changes in recorded video images of human skin surfaces. This method is termed *remote photoplethysmography (rPPG)* and can be achieved with consumer-level cameras (e.g., a webcam or mobile camera). The goal of the present publication is two-fold. First, we aim to organize future rPPG software developments in a tractable and nontechnical manner, such that the public gains access to a basic open-source rPPG code, comes to understand its utility, and can follow its most recent progressions. The second goal is to investigate rPPG’s accuracy in detecting heart rates from the skin surfaces of several body parts after physical exercise and under ambient lighting conditions with a consumer-level camera. We report that rPPG is highly accurate when the camera is aimed at facial skin tissue, but that the heart rate recordings from wrist regions are less reliable, and recordings from the calves are unreliable. Facial rPPG remained accurate despite the high heart rates after exercise. The proposed research procedures and the experimental findings provide guidelines for future studies on rPPG.

**Electronic supplementary material:**

The online version of this article (10.3758/s13428-019-01256-8) contains supplementary material, which is available to authorized users.

Imagine a situation in which physical states of people can be inferred from surveillance camera footage. Although this may sound like science fiction, the truth is that cameras can capture subtle cues about a person’s physiology that are invisible to the human eye. More specifically, progressions in the field of image processing have led to the development of algorithms that enable the extraction of the timing of heart beats from distant camera recordings of an individual’s skin. This novel method is termed *remote photoplethysmography (rPPG)*. Here we describe a study on its accuracy in detecting heart rates in a variety of conditions and we provide guidelines for future investigations into rPPG’s applicability and effectiveness.

## Nonremote photoplethysmography (PPG)

Before we explain rPPG mechanisms, we first would like to give credit to a large body of preceding studies that have eventually led to the development of remote heart rate recordings. It all started with a scientific breakthrough by Hertzman and Spealman (1937). They discovered that heartbeat-induced changes in blood perfusion in skin surface can be detected by measuring changes in both diffuse light reflection off and transmission through body parts. A year later, Hertzman developed a photoplethysmograph that could measure changes in an individual’s heart rate over time (Hertzman, 1938). The modern variant of photoplethysmography (PPG) consists of a pulse-oximetry device that is, in most cases, clipped on an individual’s finger. A standard pulse oximeter probe emits red and infrared light that is diffusely reflected from and transmitted through skin tissue. The heart stroke volume induced pulse wave travels along the arterial vascular network, which causes changes in blood volume, and this in turn causes changes in blood oxygenation and tissue pulsations in the capillary beds of skin tissue (Kamshilin et al., 2015; Nijboer, Dorlas, & Mahieu, 1981). Because these two factors affect light scatter and absorption, changes in infrared luminance levels—targeted at capillary beds relatively close to the skin’s surface—can be used to infer how many heart beats were present within a certain time window.

## Limitations to the application of PPG

Modern medicine and many other fields rely on PPG in monitoring a patients HR. Photoplethysmography is used to detect abnormalities in a person’s physiological state—for example, by measuring heart rate or respiration (Allen, 2007). Heart rate measurements with PPG may also provide information about a person’s emotional responses (Critchley et al., 2005) or level of stress (Bousefsaf, Maaoui, & Pruski, 2013; Kranjec, Beguš, Geršak, & Drnovšek, 2014; McDuff, Gontarek, & Picard, 2014). Although the number of applications is extensive, PPG’s contact requirements limit its applicability. First, heart rate can only be measured as long as the person does not move the PPG device because movement severely distorts measurements. These movement constraints limit PPG’s use during sports and other activities that require individuals to move freely. Second, the attachment of a pulse oximeter to a body part draws attention to the measurement, making users aware of that they are being monitored. In psychology, it is often preferred that participants remain naïve about the measurements to prevent that they consciously or unconsciously influence their heart rate and other outcomes. Luckily, the noncontact, remote version of PPG is not limited by the above-mentioned issues.

## Remote PPG

RPPG, also known as *imaging PPG* (iPPG or PPGI) or *noncontact PPG* (ncPPG), is based on the same principle as PPG. The difference is that rPPG remotely records changes in blood perfusion. It basically consists of digital camera recordings of variations in light reflected from skin tissue. Its first application is described in Wieringa, Mastik, and van der Steen (2005). Using a remote camera and red-to-infrared light-emitting diodes, they found pulsatile variations in luminance at the same rate as the heart’s pulse across the recorded image frames of human skin surface of wrists. A couple of studies followed rapidly, replicating and improving the method with relatively complex, custom-made apparatus (Cennini, Arguel, Akşit, & van Leest, 2010), and infrared-sensitive cameras (Humphreys, Ward, & Markham, 2007; Zheng, Hu, Chouliaras, & Summers, 2008). Only after Verkruysse, Svaasand, and Nelson (2008) had demonstrated that accurate heart rate measurements can be achieved with an affordable, consumer-level camera and “normal” ambient light conditions did rPPG become more popular. Frankly, it is an appealing phenomenon that heart pulsations in the skin are not visible to the human eye but can be recorded by a simple webcam. Since this finding, numerous studies have tested rPPG under ambient light conditions—for example, showing that rPPG in combination with face tracking allows heart rate measurements from multiple people at the same time with minimal motion distortions (Poh, McDuff, & Picard, 2010; Wang, Stuijk, & De Haan, 2015).

## rPPG’s underlying physiological mechanism and algorithms

What does rPPG actually measure? The basis of the signal is fluctuations over time in reflected luminance from a skin surface. Simply put, the camera-recorded luminance values fluctuate as a function of every heartbeat. Most recent models suggest that the luminance fluctuations are caused by changes in capillary tissue movement (Daly & Leahy, 2013; Kamshilin et al., 2015). These luminance changes are so small that human perception cannot detect them. Under proper illumination conditions, a camera sensor can detect these fluctuations, which can be extracted by the application of several signal-processing steps, including filtering, independent component analyses, and other data-processing approaches (for reviews, see Rouast, Adam, Chiong, Cornforth, & Lux, 2018; Sun & Thakor, 2016). In many scientific publications about rPPG, the signal processing steps are described and then benchmarked on a variety of videos, mostly recorded from human faces. However, the developed algorithms and software codes in which these processing steps are implemented have so far not been made available to the public. Here it is our main goal to implement the most basic rPPG signal processing steps in a code that is available to the public.

## Present study

To achieve this main goal, we have created rPPG software available to everyone, to increase the applicability of the rPPG method by offering this accessible and free software. The license under which this software is released allows others to further develop the software for scientific and public use. Please note that it is not our intention to develop a state-of-the-art rPPG algorithm that produces better results than previous algorithms. This means that the here-described processing steps are standard and described in most rPPG publications.

Our second goal was to write a manuscript for a broad audience, beyond clinical and technical fields. Although rPPG is a promising utility in numerous applications, mainly in clinical settings (Aarts et al., 2013; Klaessens et al., 2014; Tarassenko et al., 2014), it has not yet been embraced by other scientific fields that are interested in the relationship between heart rate, behavior, and cognition (but see Bousefsaf et al., 2013; Kwon, H. Kim, et al., 2012; McDuff, Estepp, Piasecki, & Blackford, 2015; McDuff, Gontarek, & Picard, 2014). We aim to describe the rPPG most basic processing steps in layman terms such that it can also be understood and tried out by scientists that work outside the technical areas of computer science, informatics, and mathematics.

The third goal of this article was to guide rPPG research toward a standardized procedure to test and report rPPG’s accuracy in a variety of conditions relevant to most sciences. We noted that many articles on rPPG use different analyses to benchmark their algorithms. We therefore aimed to provide several basic analyses that are needed to provide at least the most relevant information about an rPPG algorithm’s accuracy.

In line with our first two goals, we developed a test procedure that assesses rPPG in as broadly applicable a context as possible. This involves a different approach than previous studies have pursued, for we prioritized usability over state-of-the-art methodology:

(i) A consumer-level webcam was used, because this hardware is available to most people.
(ii) The software should be applicable to any type of skin surface on any part of the body. As far as we know, rPPG’s accuracy with consumer-level cameras, of which we define the maximum specifications as 1080p resolution and 60 frames per second, has only been reported for video recordings of faces. Verkruysse et al. (2008) mentioned that they tested rPPG on the legs and arms, but they did not report any results. Other studies have tested rPPG with higher-end cameras on the hands (Kviesis-Kipge & Rubīns, 2016; Marcinkevics et al., 2016; Rubins, Miscuks, Rubenis, Erts, & Grabovskis, 2010; Sun, Hu, Azorin-Peris, Kalawsky, & Greenwald, 2013) or with a green-colored light source (Teplov, Nippolainen, Makarenko, Giniatullin, & Kamshilin, 2014). Since it is possible that the facial skin surface is minimally visible, either due to head orientation or privacy reasons (e.g., faces are blurred or blocked), it is important to also examine rPPG’s accuracy on body parts other than faces. Hence, we tested rPPG’s accuracy on the skin surface of the arm (wrist and hand palm) and leg (calf), which both are body parts that are most likely visible in any type of video recordings of humans. Furthermore, we expected that the pulse signal would be weak in the calves, because of the small amount of superficial blood vessels in the calf’s skin. This would allow us to benchmark rPPG in a challenging condition.
(iii) Individuals could have variable heart rates during recordings, especially when in a state of arousal due to stress experiences or other psychological and physical demands. Because variability might affect rPPG’s accuracy, its effects should be taken into account. Recent studies have tested rPPG accuracy both after and while participants performed exercise. However, these studies had several limitations, such as a narrow range of exercise conditions (Sun et al., 2011; Yan et al., 2017), the absence of statistics comparing accuracies between exercise conditions (Poh & Poh, 2017; Sun et al., 2011; Wang, den Brinker, Stuijk, & de Haan, 2017b; Yan et al., 2017), and the sole focus on facial measurements (Poh & Poh, 2017; Sun et al., 2011; Wang, Balmaekers, & de Haan, 2016; Wang et al., 2017b; Yan et al., 2017). We assessed rPPG’s accuracy under conditions in which participants either were at rest or had higher and more variable heart rates, after exercise.
(iv) When participants reach heart rates above approximately 100 beats per minute (BPM), the respiration rate can rise to a level that is similar to the heart rate at rest. When no prior knowledge about the individual’s physical state is available, it can be difficult to dissociate heart rate signals from respiration signals, especially when the breathing rate dominates the signal variance or when the pulsatile variations are highly distorted by noise. Studies often report the presence of respiration signals within the recorded heart rate signals, but no simple and accessible solution has so far been provided to filter out the signal and select heart rate rather than respiration for analysis. Here we implemented a straightforward decision rule that allowed us to dissociate heart rate and breathing rate in the signal’s frequency spectrum.

Our third goal, to develop a basic, standardized report procedure that would assess rPPG accuracy from several perspectives, was achieved by reporting correlations and difference scores between rPPG and a reference, and by displaying scatterplots and Bland–Altman plots for qualitative inspection of rPPG’s accuracy and the linearity of its relationship with the reference. RPPG’s correlations with the reference are also reported as a function of video length, to inspect how much recording time was needed to reach a preferred level of accuracy (Tulyakov et al., 2016). Finally, rPPG heart rate measurements might correlate with the reference’s measurements, but the correlations could be too weak to determine whether or not a person has exercised. Thus, rPPG’s accuracy in dissociating between exercise-induced differences in heart rates per body part is reported, in the form of difference scores and signal detection theory’s calculation of the distinctiveness of distributions (area under the curve, or AUC).

## Method

### Participants

Twenty-one individuals participated in the experiment (age *M* = 24.24 years, *SD* = 5.77; 11 male, 10 female). All participants received study credit or money for participation, were naïve to the purpose of the experiment, gave informed written consent before the experiment, and were debriefed after the experiment. To ensure good skin visibility, the participants had no skin makeup and wore loose clothing that could be easily rolled up for recordings of the legs and arms. The participants’ appearances varied considerably. Some of them had facial hair or wore glasses. Skin tone ranged from pale white to dark brown. Since the performance of physical exercise was part of the experiment, individuals could only participate when they stated that they had no medical heart condition. The experiments conformed to the ethical principles of the Declaration of Helsinki and were approved by the local ethics commission of Utrecht University.

### Design and apparatus

**Fig. 1:**
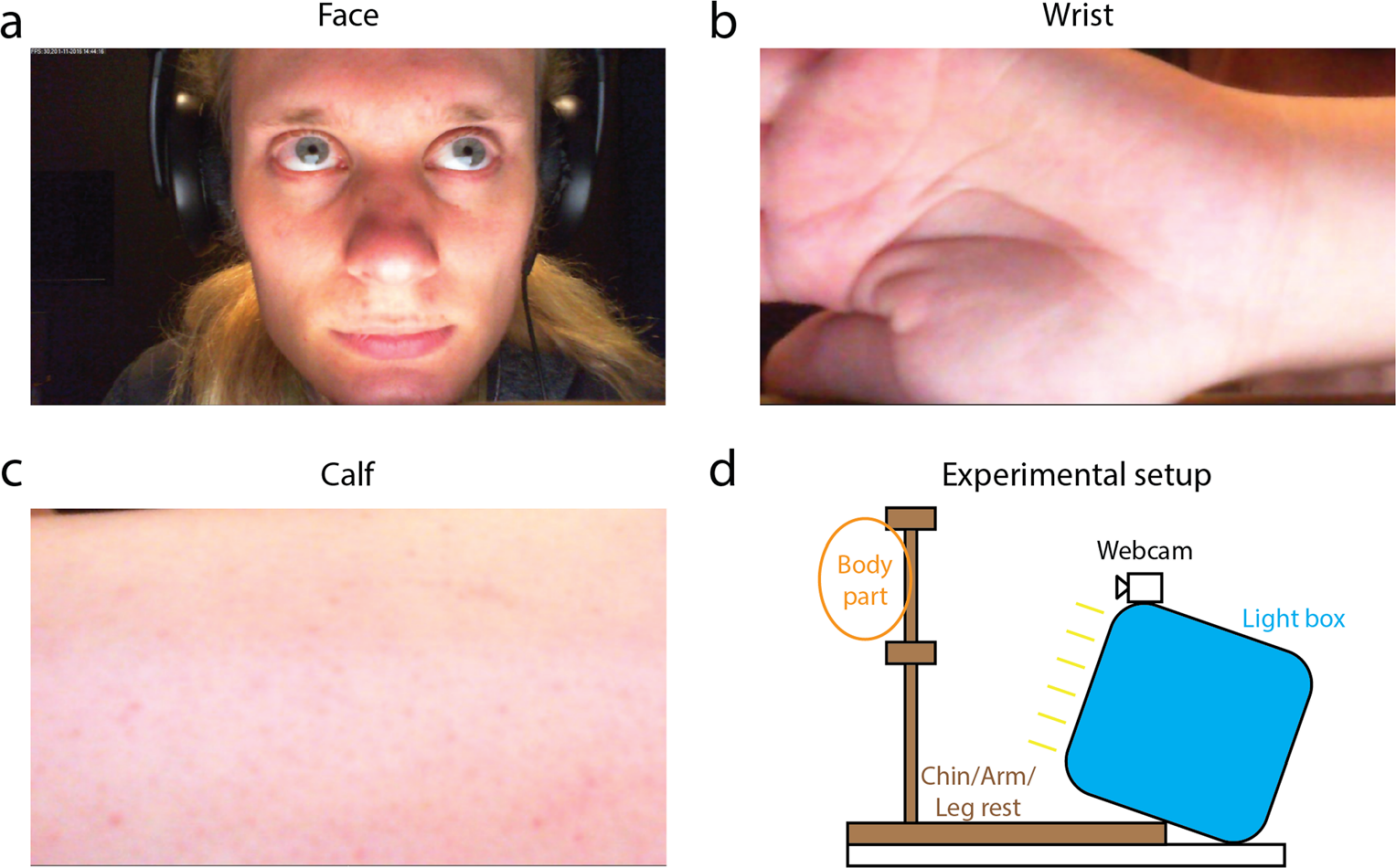
Snapshots from recordings of the face (a), wrist (b), and calf (c) of author K.v.d.K. The apparatus consisted of a wooden structure that supported the body parts to minimize movement, a webcam for recordings, and a light box for equal illumination across the surface of the body parts

The experiment consisted of a repeated two-factor design with the independent factors of exercise and body part recording. The exercise conditions consisted of rest, light exercise, and moderate exercise, and the recorded body parts were full faces, wrists including the palm of the hand, and calves, covering the entire backside of the lower leg from ankle to knee (see Fig. 1a–c).

Videos were recorded in a room with ambient background lightning by fluorescent TL tubes. Participants sat on a chair in front of a camera (Fig. 1d). The camera was a low-end LifeCam HD-3000 webcam manufactured by Microsoft (Redmond WA, United States) that recorded uncompressed AVI videos at 30 frames per second with a resolution of 1,280 × 720 pixels at eight-bit quality per RGB channel. Note that the resolution and frame rate of the camera might not necessarily affect rPPG’s accuracy (Blackford & Estepp, 2015). Videos were recorded with the open-source program iSpy (http://www.developerinabox.com/). Default settings for brightness, contrast, saturation, sharpness, and white balance were used, and all automated dynamical corrections were turned off. Exposure, a parameter that sets the duration over which light is captured by the webcam’s CCD per frame, was set at – 10. All other amplification and control options in iSpy were turned off. The camera was placed 20 cm from the body parts. A light box, placed at the same distance, illuminated the body parts with 1,370 cd/m^2^. These settings ensured proper illumination conditions and prevented saturated regions in the image frames. Facial pixel values were 208, 150, and 136, averaged across all facial pixels, then averaged across video frames, and then averaged across all videos, per RGB channel, respectively.

A standard pulse oximetry (contact PPG) finger-clip device, the CMS50E manufactured by Contec (Qinhuangdao, China), was used for the reference heart rate measurements. The pulse oximeter was attached to the right index finger and connected to a desktop computer through an USB cable. Custom made MATLAB (MathWorks, Natick, MA, USA) software recorded the heart rate pulses from the oximeter in parallel with the video recordings. The pulse oximeter was not attached during exercise.

### Procedure

#### Rest condition

Participants first rested for a couple of minutes in a chair (rest condition). Then, a sequence of three recordings were made from the participant’s head, wrist, and calf. Participants were instructed to position themselves as stable as possible in front of the camera and to minimize movement during the recordings. The recorded body parts were placed inside a wooden support structure in front of the camera (see Fig. 1d). The order of body part recordings was randomized and counterbalanced across participants. Each recording lasted approximately 30 s.

#### Light exercise

Next, participants had to exercise by running on the spot (i.e., making running motions while staying on the same exact location). The moment the researcher K.v.d.K. heard a substantial increase in breathing rate (approximately after 60 s), the same recording procedure described above was performed. Participants were asked to exercise for a short moment between each recording to keep their heart rate at relatively the same level across recordings.

#### Moderate exercise

After the light exercise and after the second sequence of recordings, participants performed the running exercise again, but this time longer than in the previous light exercise condition (approximately 120 s). If participants reported fatigue after running for a while, they could switch to performing jumping jacks (i.e., moving both arms and legs in and out in parallel while jumping). Again, recordings were made from each body part after the exercise.

### Software development

The extraction of the heart rate signal from videos of human skin surface requires complicated image processing software. Before we explain how this can be accomplished, we want to note that we have made our MATLAB software and supporting details available to the public on https://github.com/marnixnaber/rPPG. We also invite others to either edit and improve these scripts or write custom software and send their scripts to us for benchmark testing. Improved versions of the algorithm published in this article will fall under an open-source GNU general public license (see the website above for details). Either before or after publication of new rPPG software, scientists can contact author MN to request to test the performance of their rPPG software on a set of videos recorded under variable conditions. This software remains intellectual property of the owner and it will not be published on the website without permission. Only the test reports will be published on a webpage (http://www.marnixnaber.nl/rPPG/). The goal is to gradually extend the set of videos in the future by including more video recordings made with a large variety of apparatus that differ in cameras, object distance, FPS, resolution, lighting conditions, skin colors, and so forth. These videos will not be made publicly available, because of privacy and to prevent the development of overfitting algorithms (i.e., generalization errors). In other words, the precise content of the test videos will remain unknown to prevent that participants are recognized and that software is adapted in such a way that it can only measure heart rate accurately for this set of videos but not for other videos. The test results will be made available in summary format on the aforementioned webpage that provides an overview of all available rPPG software and corresponding heart rate detection performances.

### Analysis

Webcam-based rPPG relies on a series of image-processing steps to extract blood pulsation from the recorded videos and to determine the heart’s beating rate (HR). These steps consisted of (i) spatiotemporal cropping of videos, (ii) facial skin selection, (iii) averaging and filtering signals, (iv) independent component analysis, (v) fast Fourier transform, (vi) filtering power spectra, and (vii) respiration/movement signal rejection. Here below we provide detailed information per individual processing step.

#### Spatiotemporal cropping

Heart beat-induced fluctuations in reflectance can only be detected at the skin’s surface. Therefore each video was cropped to a fixed region of interest, removing irrelevant background objects. Faces were automatically detected with a cascade object detector of MATLAB’s computer vision system toolbox. Videos were also cropped in time by removing the first and last 3 s, because the first part of the video often contained an increase in the camera’s light sensitivity and the second part tended to contain more body movements, as participants anticipated the end of recording.

#### Facial skin selection

**Fig. 2:**
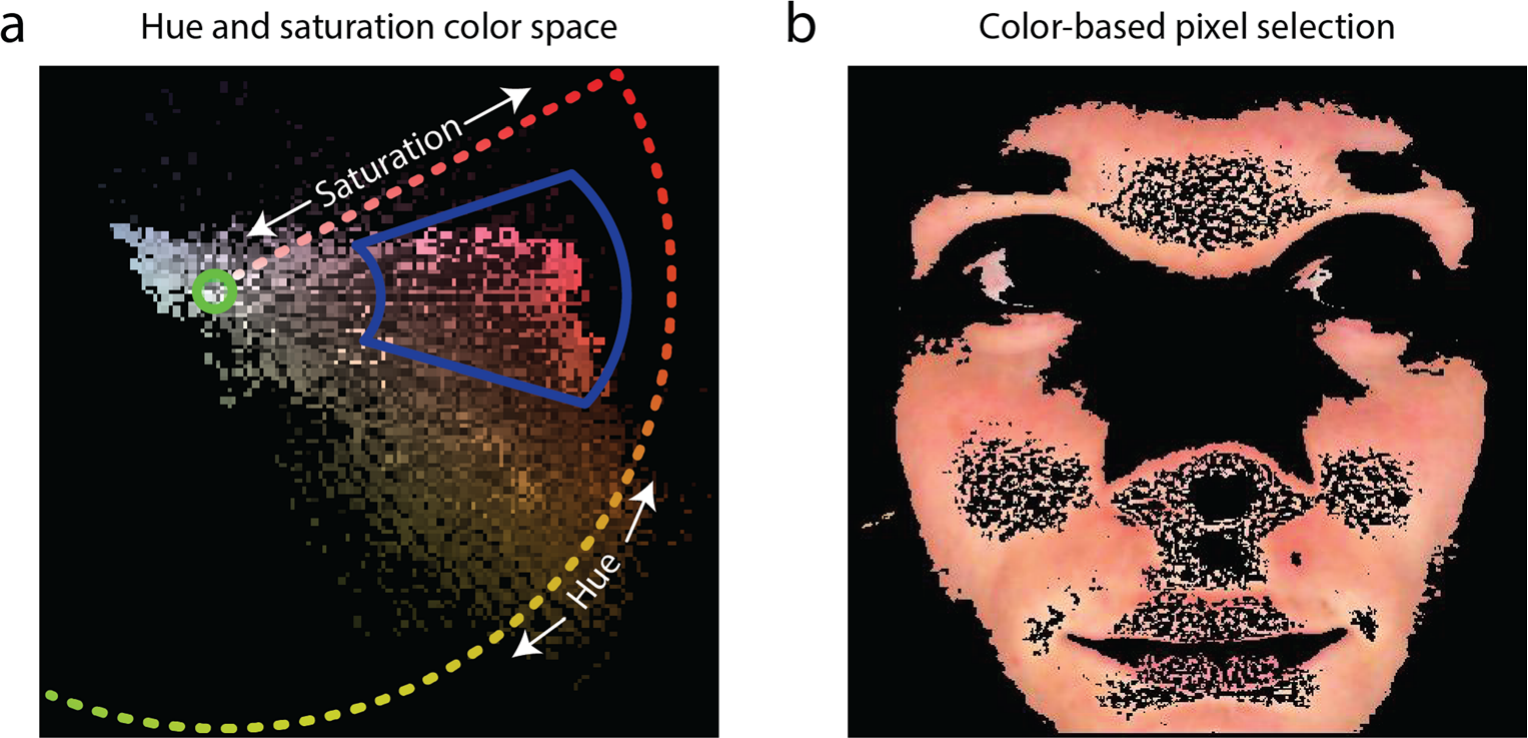
Example color space, showing pixels from a single frame from a face recording (a). Hue is circularly represented as a function of angle around the color space center (green circle), and saturation is radially represented as a function of eccentricity extending from the colorless center. The blue wedge indicates which pixels in the hue–saturation color space were selected for rPPG processing. The skin color selection procedure ensured that the processed pixels only represented the skin surface and not eyes, clothes, or other nonskin areas (b)

The background, clothing, teeth, hair, and other irrelevant parts were filtered out of each frame with a skin color selection procedure. Our script offers to methods to detect the skin: (1) automatic selection based on color clusters, and (2) manual selection of hue and saturation ranges. The automatic selection consisted of a *k*-means clustering approach (squared Euclidean distance, four clusters, maximum of 100 iterations) on *a* and *b* dimensions of CIE LAB color space divided the area within a bounding box around the face in separate color clusters. The color cluster with the most pixels in the center of the face was selected as the skin pixels. The manual selection consisted of the selection of pixels that fell within a range of skin hues and saturations. This range was set for the first frame and then used for the following frames of each video. The hue and saturation ranges were set manually by researcher K.v.d.K. by adjusting the size and angle of a selection wedge within the hue–saturation color map (Fig. 2a). For example, see Fig. 2b for the selected pixels of the first frame of a face with hues and saturation levels that fell within the wedge.

#### Averaging and filtering signals

**Fig. 3:**
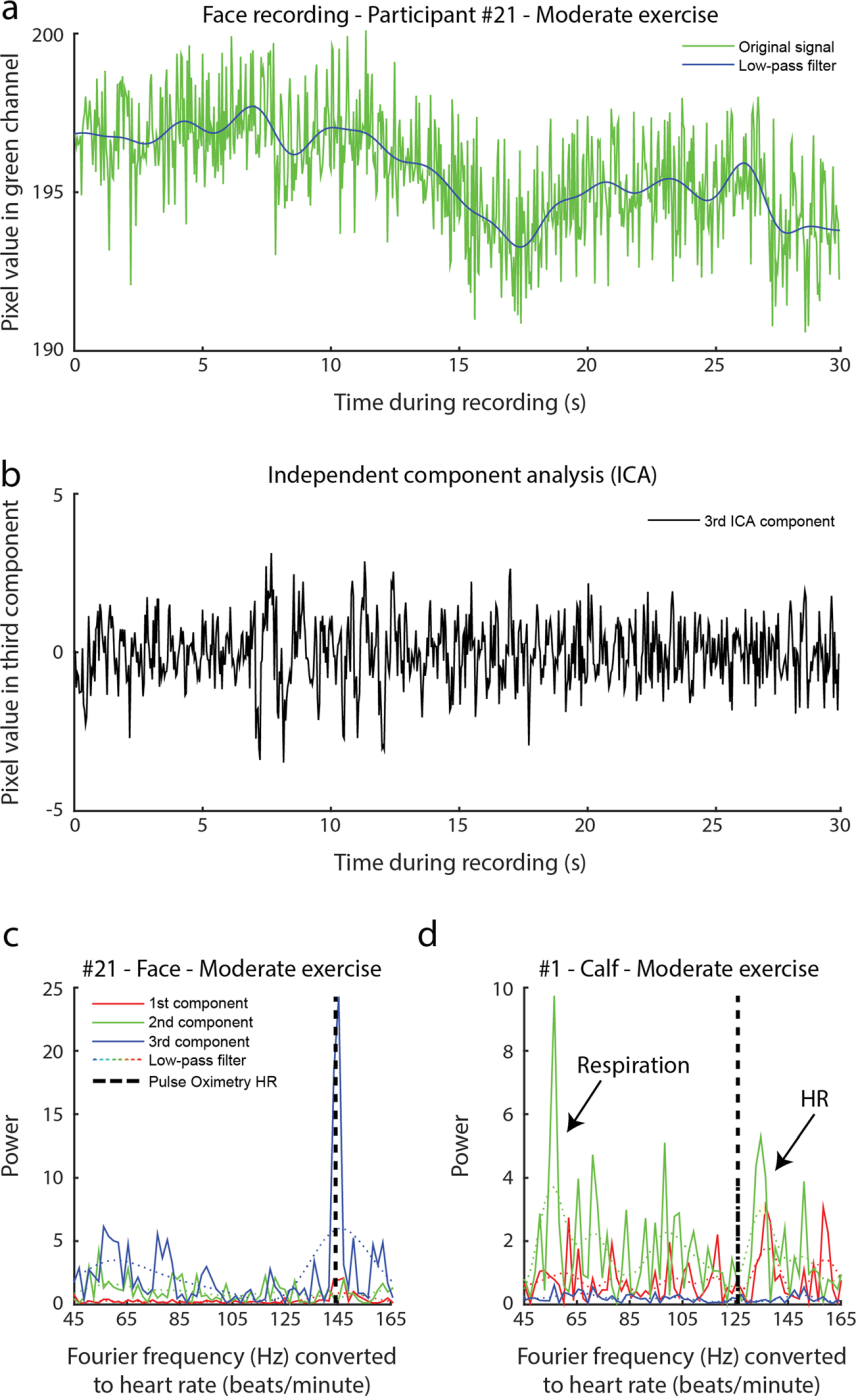
(a) The jagged solid line represents an example of the original signal of pixel values of the green channel of a video recording of a face after moderate exercise. The blue line is a low-pass filter of the original signal. The low-pass signal was subtracted from the original signal, to remove slow fluctuations due to movement and other confounding factors. Next an, an independent component analysis (ICA) was performed. (b) The strongest component computed from the ICA. Power frequency spectra were computed from the resulting component signals. (c) Example of a frequency spectrum. Fast Fourier transform low-pass filters (dotted lines) were applied to the spectra, to remove noise and highlight a multitude of individual power peaks that appeared close together within a small range of varying heart rates. (d) Sometimes the respiration signal power was strongly present in the frequency spectrum. In these cases, the second-highest power peak was selected as the corresponding heart rate. The black dashed lines in panels c and d indicate the reference heart rate measured with the pulse oximetry device

The average of all selected pixels was computed per video frame and RGB color channel. The resulting average pixel value as a function of time was noisy (green line in Fig. 3a) and subject to considerable low-frequency variations. To remove any influence of movement and other factors inducing low-frequency changes in the signal, a zero-phase sixth-order Butterworth filter with a cutoff frequency setting of 0.04 Hz was applied to the raw signal, to compute the low-frequency signal (blue line in Fig. 3a). This signal was computed per RGB channel and subtracted from the raw signal. The resulting signals fluctuated around zero and contained no low-frequency fluctuations (Fig. 3b).

#### Independent component analysis

The filtered signals from each RGB color channel were used as input for an independent component analysis (ICA; Comon, 1994), to extract the most prominent component signal present in all color channels (i.e., most likely the heart rate), thereby increasing the signal-to-noise ratio. Performing the ICA is useful for improving heart rate signal extraction (Holton, Mannapperuma, Lesniewski, & Thomas, 2013). The ICA looked for three components, using a maximum of 2,000 iterations, with verbose set off and stabilization turned on.

#### Fast Fourier transform (FFT)

The component signals were fast Fourier transformed. An FFT converts the component signals into an estimation of power spectra (squared magnitude) that indicates which oscillatory sine-wave frequencies were represented most powerfully in each component signal (Fig. 3c). A high peak in power at a certain frequency means that the component was made up mostly of a sine-wave at that specific frequency. This frequency is in most cases a reflection of the detected heart rate. For convenience, we represented power as a function of heart rate rather than frequency. Previous studies had applied a time–frequency analysis to show how the frequency spectrum changes as a function of recording time (Hu, Peris, Echiadis, Zheng, & Shi, 2009), but the short-time Fourier transform provided no clear heart rate signal with the present data, probably due to the relatively short recordings and low signal-to-noise ratio in many videos.

#### Filtering power spectra

Heart rate tends to decrease toward a baseline rest rate after exercise. This causes the power peak representing heart rate in the frequency spectra to be smeared out or appear as small individual peaks in close proximity around a range of heart rate frequencies. To be able to select the correct power peak at the average corresponding heart rate, and not an irrelevant power peak, the power spectra were filtered with a zero-phase third-order low-pass Butterworth frequency filter (LFF) with a cutoff frequency setting of 0.2 Hz (see the dotted lines in Fig. 3c and d).

#### Respiration/movement signal rejection

The heart rate at the highest power peak across components was selected as the final rPPG heart rate. However, it was noticed that often two relatively high power peaks were visible in the frequency spectra of the components after exercise. Often a high power peak was present below a frequency of 90 beats per minute (BPM), and a second, lower power peak was present above 90 BPM (see, e.g., Fig. 3d). In such cases, the high peak at the lower frequency was probably caused by respiration or bodily movement, while the smaller peak at the higher frequency was caused by heart pulsations (Hu et al., 2009). To autonomously extract the heart rate signal rather than other, irrelevant signals, we implemented a custom power peak selection rule consisting of two IF/THEN/OTHERWISE statements: (i) If more than two peaks were present in a single power spectrum, including one large peak below and one smaller peak above the cutoff rate of 90 BPM, and (ii) if the lower peak was not smaller than 70% of the height of the highest peak, then select the lower peak’s frequency as the heart rate. Otherwise, select the frequency of the highest power peak for the corresponding heart rate. We refer to this selection rule as *respiration rejection* (Resp). Multiple cutoff rates and minimal peak differences were explored, and the parameters described above resulted in the best correspondence between reference pulse oximetry-based heart rates and rPPG heart rates.

## Results

### Pulse oximetry and rPPG HR measurements per exercise and body part condition

**Fig. 4:**
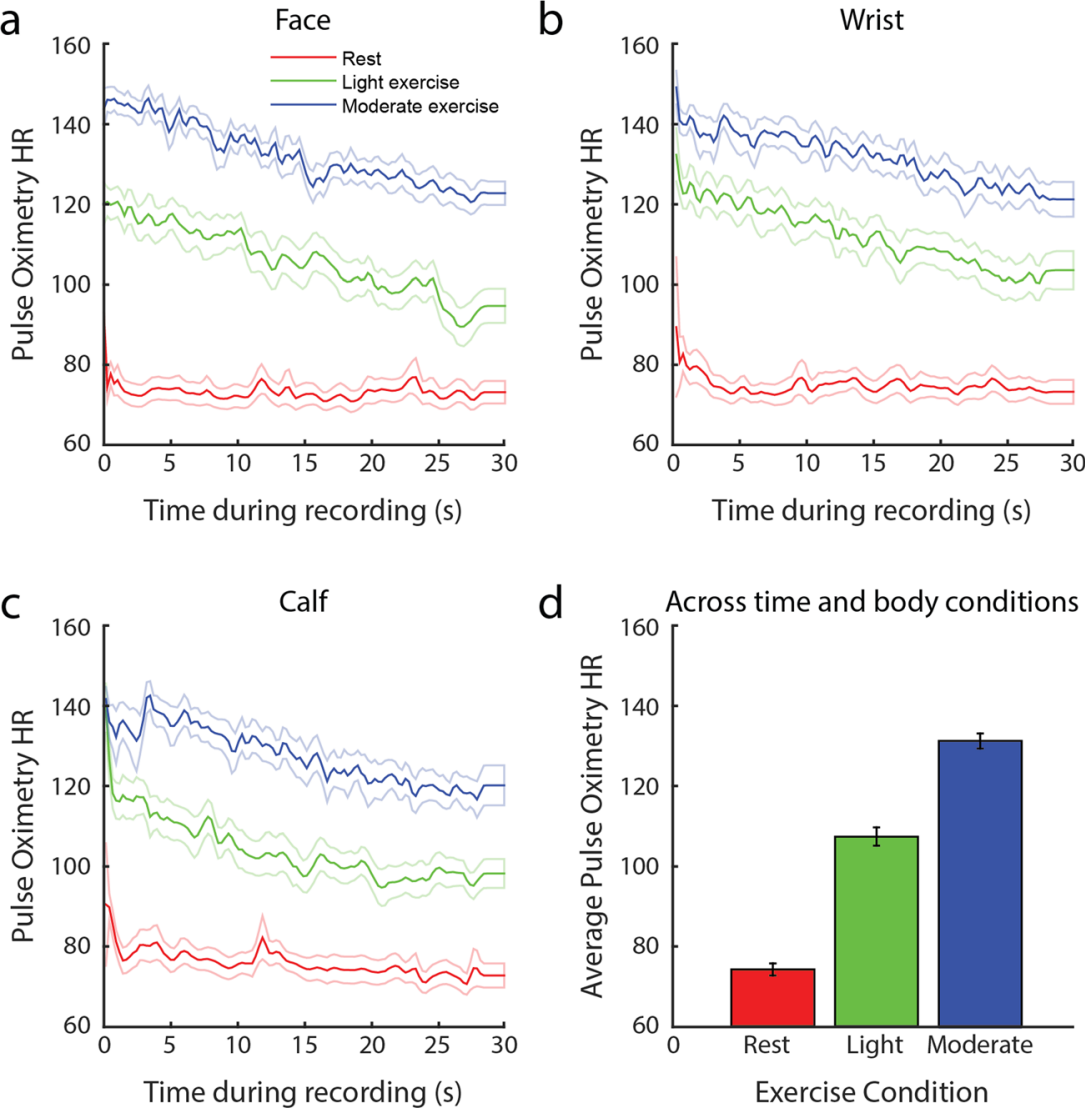
Pulse oximetry-based heart rates, in beats per minute (reference) as a function of time during rest (red), after light exercise (green), and after moderate exercise (blue), during facial (a), wrist (b), and calf (c) recordings. Average pulse oximetry-based heart rates during recording per exercise condition, pooled across all body recordings (d). The dotted lines (a–c) and error bars (d) around the mean indicate standard errors

We first performed a sanity check to ensure that the exercise instructions indeed resulted in significant differences in heart rates across exercise conditions, as measured with the reference pulse oximeter. As is shown in Fig. 4a–c, light exercise resulted in higher heart rates than at rest, and moderate exercise resulted in even higher heart rates than did light exercise. The heart rates averaged across recording durations (Fig. 4d) differed significantly across the exercise conditions [*F*(2, 20) = 259.41, *p* < .001]. Post-hoc *t* tests comparisons indicated that each exercise conditions differed significantly from the others in heart rate [rest vs. light: *t*(20) = 12.63, *p* < .001; rest vs. moderate: *t*(20) = 20.79, *p* < .001; light vs. moderate: *t*(20) = 11.16, *p* < .001]. Thus, the exercise instructions resulted in the expected increases in heart rate.

### Comparison between pulse oximetry and rPPG

**Fig. 5:**
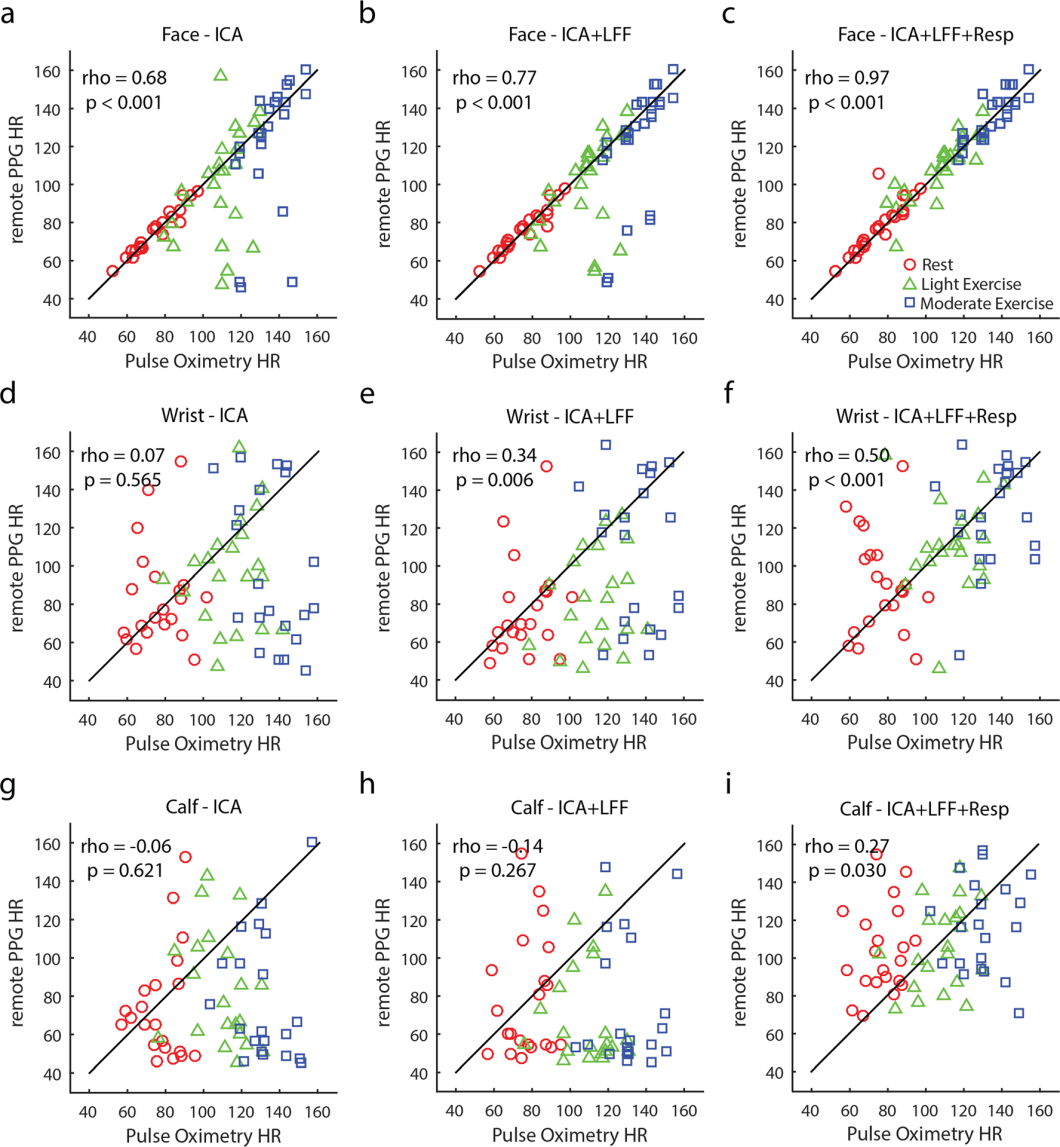
Scatterplots displaying correlations between the average heart rate (beats/min) measurements of webcam-based remote PPG and pulse oximetry-based PPG per body part (rows) and per analysis method (columns). A combined procedure of applying an independent component analysis (ICA), low-pass frequency filtering (LFF) the power spectra, and rejecting the respiration signal (Resp) provided the best correlations for all body part recordings (c, f, and i). Videos of the webcam recordings of the face provided higher correlations with the pulse oximetry recordings than did the wrist recordings, and wrist recordings were better than the calf recordings, independent of the applied analyses (compare the rows)

**Fig. 6:**
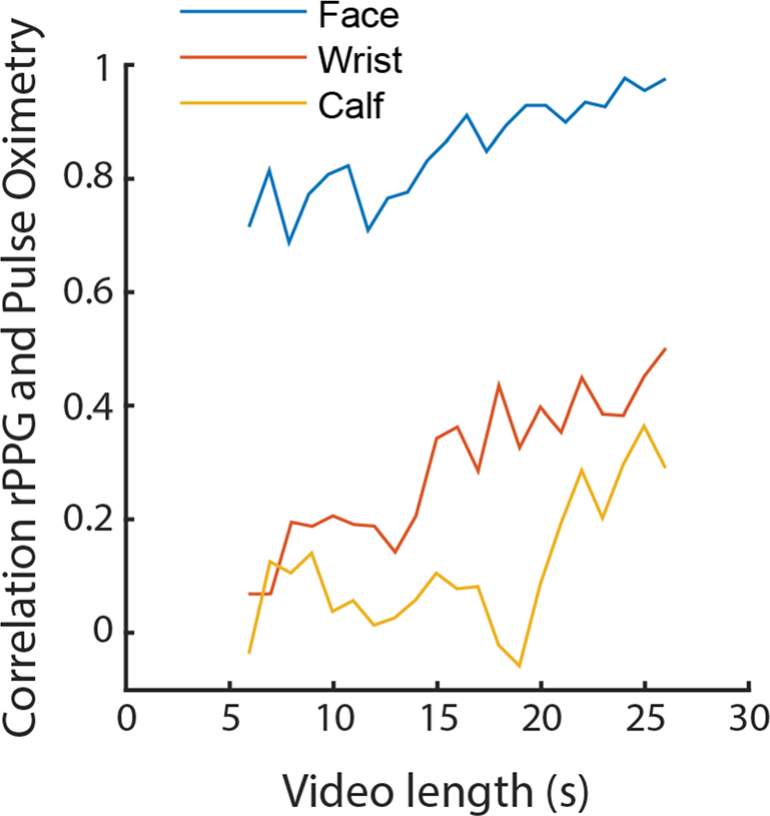
Correlations between remote and pulse oximetry-based PPG as a function of video length per recorded body part (with all filters applied: ICA + LF + Resp). Video length, on the *x*-axis, means that the data were analyzed in the period from the sixth second until the *x*th second of the video. Note that the first and last 3 s of the video were not analyzed (see the Method section), and correlations can only be calculated with a minimum of three data points

The varying exercise conditions resulted in a large range of heart rates, as measured with pulse oximetry across conditions and participants. Next, we examined whether these heart rates were comparable to the rates measured with camera-based rPPG. We calculated and display Spearman correlations in Fig. 5, per body part recording (rows) and per analysis method applied (columns). Qualitative assessment of these correlations suggested that the application of an LFF of the spectrum (see Fig. 3c and d) and respiration rejection filter produced better correlations (for Bland–Altman plots, see Supplementary Fig. S1). The heart rate measurements of facial rPPG were highly comparable to those from pulse oximetry (*r* = .97, *p* < .001), and correlations in the wrist (*r* = .50, *p* < .001) and calf (*r* = .27, *p* < .001) measurements were significantly positive but weak. The correlations between the camera-based and pulse-oximetry-based heart rate recordings depended on the amounts of video frames analyzed (Fig. 6). As more frames were added in the rPPG analysis, the correlations increased.

Next, we quantitatively assessed differences in overlap between the rPPG and pulse oximetry heart rate measurements across conditions when all filters were applied (Fig. 5c, f, and i). We performed a two-way repeated measure analysis of variance (ANOVA) on the absolute (rectified) difference between the heart rates of both measures, with the factors exercise and body part condition. A significant main effect of body part [*F*(2, 40) = 19.62, *p* < .001] and an overall inspection of post-hoc *t* tests (see Table 1 for the means and standard deviations, and Table 2 for statistical comparisons) indicated that facial rPPG was significantly more accurate than rPPG on the wrists and calves, when assuming that the reference pulse oximetry measured ground truth. A significant main effect of exercise [*F*(2, 40) = 4.54, *p* = *.*017] and a significant interaction between exercise and body part [*F*(4, 80) = 3.46, *p* = *.*012] showed that facial rPPG at rest produced the best heart rate recordings, whereas the wrist and calf recordings showed no noteworthy differences across exercise conditions.

**Table 1:** Means and standard deviations of absolute difference between rPPG and pulse oximetry heart rate (beats/min)

Exercise Body Part	Rest	Light	Mod
Face	2.34 ± 1.51	5.91 ± 4.78	5.60 ± 4.00
Wrist	29.75 ± 25.07	16.11 ± 21.03	18.78 ± 17.45
Calf	36.83 ± 24.42	19.47 ± 16.63	29.07 ± 22.63

**Table 2:** Post-hoc *t* test comparisons between differences in rPPG and pulse oximetry heart rates (beats/min)

		Face	Wrist	Calf	Face	Wrist	Calf	Face	Wrist
		Rest	Rest	Rest	Light	Light	Light	Mod.	Mod.
Wrist	Rest	4.53^***^							
Calf	Rest	6.61^***^	1.14						
Face	Light	2.28^*^	3.82^**^	6.29^***^					
Wrist	Light	2.58^*^	2.01	3.05^**^	1.89				
Calf	Light	3.83^**^	1.54	3.11^**^	3.40^**^	0.38			
Face	Mod.	1.26	4.13^***^	5.70^***^	1.03	2.37^*^	3.49^**^		
Wrist	Mod.	3.46^**^	1.57	2.56^*^	2.71^*^	0.71	0.13	3.41^**^	
Calf	Mod.	4.96^***^	0.01	1.05	4.79^***^	1.76	2.38	4.58^***^	1.71

^*^p < .05, ^**^p < .01, ^***^p < .001

Finally, we investigated whether rPPG adequately indicated which exercise condition was performed, on the basis of the detected heart rate. The average heart rates measured with rPPG, with all applied filters, differed significantly across exercise conditions [*F*(2, 20) = 29.35, *p* < .001]. Post-hoc *t* test comparisons per body part recording suggested that heart rate differed significantly across all exercise conditions for the face recordings, and that it differed both between light and moderate exercise and between rest and moderate exercise for the wrist recordings. However, heart rate did not differ across exercise conditions for the calf recordings (see Table 3). Signal detection analysis of the AUCs indicated that the heart rates measured with face rPPG during rest were 83% (AUC = 0.92) separable from the heart rates measured during light exercise, and 100% (AUC = 1.00) separable from those recorded during moderate exercise. The heart rates measured with face rPPG during light exercise were 78% (AUC = 0.89) separable from those during moderate exercise. The AUCs for the same comparisons for wrist rPPG were 32%, 73%, and 57% (AUC = 0.66, 0.87, 0.79), respectively. The AUCs for the same comparisons for calf rPPG were 5%, 14%, and 6% (AUC = 0.53, 0.57, 0.53), respectively. In sum, face rPPG provided good exercise indications, wrist recordings provided recordings useful to detect whether participants had exercised moderately versus not at all or lightly, and calf recordings were inaccurate in determining an exercised-induced increase in heart rate.

**Table 3:** Post-hoc *t* test comparisons between average heart rates (beats/min) across exercise conditions per body part condition (with all filters applied)

Body Part Recording	Comparison	*t* Value	*p* Value
Face	Rest vs. light	10.94	< .001
	Rest vs. moderate	8.23	< .001
	Light vs. moderate	17.52	< .001
Wrist	Rest vs. light	1.32	.200
	Rest vs. moderate	3.60	.002
	Light vs. moderate	3.69	.002
Calf	Rest vs. light	0.13	.895
	Rest vs. moderate	0.10	.924
	Light vs. moderate	0.05	.964

## Discussion

This study targeted the development and publication of basic open-source rPPG software and aimed to demonstrate its functionality with two experimental manipulations: (i) to investigate how accurately rPPG can detect heart rates at rest as compared to heart rates after exercise, and (ii) to examine whether rPPG targeted on calves and wrists are as accurate as rPPG targeted on faces under ambient light conditions. As far as we know, rPPG’s accuracy had not yet been reported by previous studies with a similar combination of experimental manipulations, a consumer-level camera, a relatively simple method, and an open-access rPPG algorithm.

We showed that rPPG can detect heart rates in faces slightly more accurately when the heart rate is slow (< 90 BPM) than when it is fast. It is possible that the variation in accuracy across exercise conditions could be related to signal distortions by breathing-induced movement. A more likely explanation is that exercise induced more variability in heart rates (compare the blue and red lines in Fig. 4a), and by definition it is more difficult to detect unstable heart rates, independent of the applied filtering methods. Nonetheless, the application of a low-pass filter on the power frequency spectra of the measured rPPG signal helped take into account variable heart rates. Future software improvements could try to cover such variabilities more accurately by measuring heart rate as a function of recording time with a sliding window over the signal (e.g., time–frequency analyses). Note that such analyses require shorter time windows, resulting in less signal power, and thus lower rPPG accuracies.

Although rPPG was highly accurate for video recordings of the face, recordings of the wrist diminished accuracy to such a degree that rPPG could only detect whether a person had performed moderate versus either light or no exercise. RPPG targeted on the calf was unreliable. An explanation for the differences in rPPG’s accuracy across body parts is that faces have a very high amount of microvascular networks in the superficial skin layers (Spalteholz, Spanner, Nederveen, & Crawford, 1967). The wrists also have many veins visible at the skin surface, but the calves lack such anatomical characteristics.

The low accuracy of rPPG measurements on the wrists and calves could be improved by applying more sophisticated rPPG algorithms and apparatus that take into account the distorting effects of bodily movements (van Gastel, Stuijk, & de Haan, 2016a, 2016b) and apply polarization camera filters (Kamshilin et al., 2016; Sidorov, Volynsky, & Kamshilin, 2016; Trumpp, Bauer, Rasche, Malberg, & Zaunseder, 2017). An interesting option would be to identify the best angle in color space along which pixel colors change as a function of heart rate rather than motion (e.g., Bousefsaf et al., 2013; Wang, den Brinker, Stuijk, & de Haan, 2017a). Note, however, that the present article’s goal was not to implement such state-of-the-art algorithms but to initiate an open-access collaborative development project that will hopefully lead to state-of-the-art algorithms and improved rPPG accuracies in the future.

One goal of the present article was to provide scientists with an open-source script that they can use to extract heart rate from videos of participants. We see several applications of our algorithm in the field of social sciences, including psychology. Heart rate and heart rate variability (HRV) are indicators of stress, workload, and emotion processing. For instance, the heart rate slows down more when people watch unpleasant stimuli than when they watch neutral or pleasant stimuli (Appelhans & Luecken, 2006, Greenwald, Cook, & Lang, 1989; Winton, Putnam, & Krauss, 1984). RPPG could thus potentially be used to determine whether or not people find advertisements and other media types pleasant. Conversely, the heart rate tends to accelerate when observing negative as compared to positive facial expressions (Critchley et al., 2005; Levenson, Ekman, & Friesen, 1990). Heart rate measurements with rPPG might thus reveal which emotions were experienced during interaction without making participants aware of the measurements. Although these possibilities have not yet been examined, studies have used rPPG to show that the components of HRV (e.g., the ratio between low- and high-frequency changes in HRV) change when participants perform a stressful task, as compared to episodes of relaxation (Bousefsaf et al., 2013; McDuff et al., 2014). These initial finding suggest that rPPG is an affordable and accessible tool to measure changes in task demands in laboratories and work-related environments.

Camera-based systems such as rPPG enable more than just the detection of heart rates. One interesting development is the detection of blood oxygen saturation by using a remote SpO2 camera setup that uses multiple wavelengths of light (van Gastel et al., 2016a; Wieringa et al., 2007). Another possibility is to record respiration rate with rPPG (van Gastel et al., 2016b). In the present study, we ignored respiration to accurately detect heart rate. The influence of respiration on the rPPG power spectra can be a problem when people have exercised and respiration rate becomes higher than 50 breaths per minute, therewith entering the range of heart rates. In other words, we treated the potential influence of respiration on rPPG purely as a confounding signal. However, it can be of great value to use rPPG to measure respiration (Sun et al., 2011; Tarassenko et al., 2014). Although this is out of the scope of the present study, future work could explore to what degree respiration is detectable in a variety of conditions. These studies should include validated measurements of respiration rates to confirm that the presence of a low frequency signal in the data is indeed caused by breathing.

In addition to the experimental investigations described above, this article was also written with the goal to improve the quality of scientific investigations into rPPG accuracy by (i) creating a standardized testing procedure for the assessment of rPPG’s accuracy and by (ii) describing a standardized report procedure that assesses rPPG’s accuracy in several manners. We hope that this article will serve as a guide for future publications on rPPG. We further would like to extend our video database of human skin recordings and invite other scientists to share existing databases with us.

This study focused on the advancement of an affordable, simple, and accessible rPPG method. However, we do acknowledge the importance and relevance of advancing rPPG methods with both high temporal and spatial resolutions. To facilitate the accuracy of our method, we utilized fast Fourier analysis (FFA) and other image-processing steps. However, FFA can potentially be inaccurate when averaging PPG waveforms across all pixels from the face’s surface, due to the possibility that not all skin pixels display a signal with the same phase (Moço, Stuijk, & de Haan, 2016; Teplov et al., 2014). Despite this limitation, we have shown that the facial measurements are still close to perfection, thus indicating that signal averaging across skin surface is not necessarily detrimental. Nonetheless, future studies could try to improve the accuracy of wrist and calf rPPG by synchronizing the phase of the heart rate signal across the skin’s surface (Kamshilin et al., 2016). Another solution would be to divide the face in multiple regions of interest (ROIs) and perform separate signal analyses per ROI (Kwon, J. Kim, et al., 2015; Po et al., 2018; Sun et al., 2011) before combining information from the most relevant ROIs.

Another limitation of the present algorithm is the setting of several parameters for the respiration rejection. It is yet unknown whether these parameters are robust and lead to comparable performances in other video recordings.

In sum, rPPG with consumer-level cameras is a promising heart rate measurement tool, at least when targeted on facial skin surfaces. This study showed that the application of rPPG on nonfacial skin surfaces is a challenge. However, computer-imaging science is progressing rapidly. Many solutions that improve the extraction of the heart rate signal from videos have recently been discovered, including the tracking of faces, the use of filters of irrelevant color and motion changes, and algorithms that detect pulsatile body movements (de Haan & Jeanne, 2013; de Haan & van Leest, 2014; Wang et al., 2015). We hope that rPPG imaging experts will continue to improve rPPG methods to become more affordable and accessible and to make their software available to the public through https://github.com/marnixnaber/rPPG.

## Electronic supplementary material

ESM 1 (PDF 377 kb)

## Data Availability

The datasets generated during and analyzed during the present study are available from the corresponding author on reasonable request. The videos generated during the present study are not publicly available, because of privacy issues and because the videos will be used to benchmark other rPPG algorithms. It is important that other algorithms do not gain access to these videos before publication, since algorithms could be adapted in such a way that they were only accurate when applied to the present set of videos (i.e., overfitting).
