# Supplementary material for: An open-source remote heart rate imaging method with practical apparatus and algorithms
Source: Behav Res Methods. 2019 May 31;51(5):2106–19. doi: 10.3758/s13428-019-01256-8 (PMC6797647; doi:10.3758/s13428-019-01256-8)
Supplement: Supplementary file 1 — (PDF 377 kb) [file 13428_2019_1256_MOESM1_ESM.pdf]

Supplementary Information

Title

Standardized procedures for the testing and reporting of remote heart rate imaging with practicable equipment and algorithms

Authors

Koen M. van der Kooij[1], Marnix Naber[1]

[1]Experimental Psychology, Faculty of Social Sciences, Utrecht University, Heidelberglaan 1, 3584CS Utrecht, The Netherlands

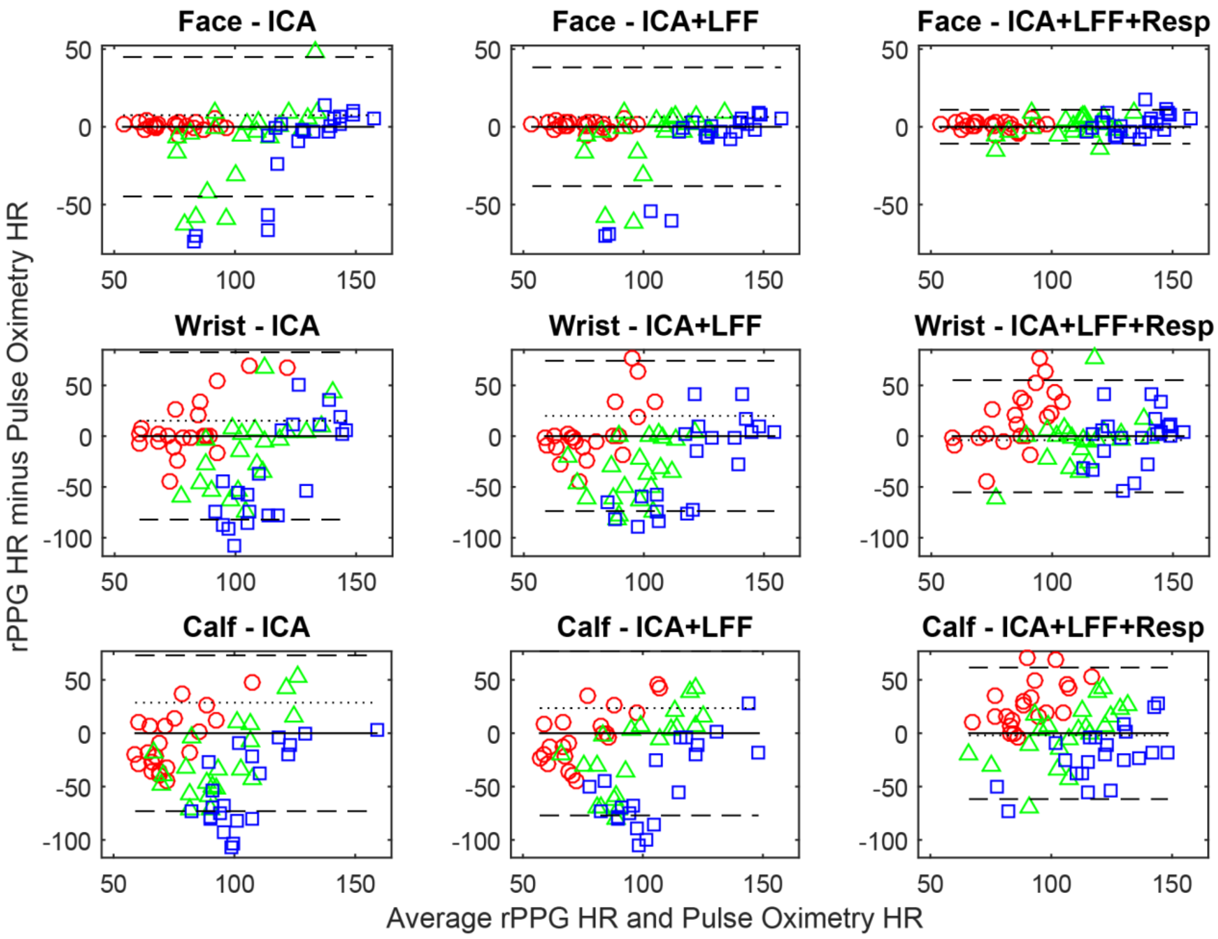

Figure S1. Alternative for scatterplots in Fig. 5. These Bland-Altman plots show the difference between rPPG and pulse oximetry heart rate measurements in beats per minute. Dotted lines indicate mean difference, dashed lines indicate the 1.96 standard deviation borders.
